# Black sheep, dark horses, and colorful dogs: a review on the current state of the Gene Ontology with respect to iron homeostasis in *Arabidopsis thaliana*

**DOI:** 10.3389/fpls.2023.1204723

**Published:** 2023-07-24

**Authors:** Hans-Jörg Mai, Dibin Baby, Petra Bauer

**Affiliations:** ^1^ Institute of Botany, Heinrich Heine University, Düsseldorf, Germany; ^2^ Heinrich Heine University, Center of Excellence on Plant Sciences (CEPLAS), Düsseldorf, Germany

**Keywords:** gene ontology, GO term, biological process, *Arabidopsis thaliana*, iron homeostasis, enrichment analysis, gene set

## Abstract

Cellular homeostasis of the micronutrient iron is highly regulated in plants and responsive to nutrition, stress, and developmental signals. Genes for iron management encode metal and other transporters, enzymes synthesizing chelators and reducing substances, transcription factors, and several types of regulators. In transcriptome or proteome datasets, such iron homeostasis-related genes are frequently found to be differentially regulated. A common method to detect whether a specific cellular pathway is affected in the transcriptome data set is to perform Gene Ontology (GO) enrichment analysis. Hence, the GO database is a widely used resource for annotating genes and identifying enriched biological pathways in *Arabidopsis thaliana*. However, iron homeostasis-related GO terms do not consistently reflect gene associations and levels of evidence in iron homeostasis. Some genes in the existing iron homeostasis GO terms lack direct evidence of involvement in iron homeostasis. In other aspects, the existing GO terms for iron homeostasis are incomplete and do not reflect the known biological functions associated with iron homeostasis. This can lead to potential errors in the automatic annotation and interpretation of GO term enrichment analyses. We suggest that applicable evidence codes be used to add missing genes and their respective ortholog/paralog groups to make the iron homeostasis-related GO terms more complete and reliable. There is a high likelihood of finding new iron homeostasis-relevant members in gene groups and families like the *ZIP*, *ZIF*, *ZIFL*, *MTP*, *OPT*, *MATE*, *ABCG*, *PDR*, *HMA*, and *HMP*. Hence, we compiled comprehensive lists of genes involved in iron homeostasis that can be used for custom enrichment analysis in transcriptomic or proteomic studies, including genes with direct experimental evidence, those regulated by central transcription factors, and missing members of small gene families or ortholog/paralog groups. As we provide gene annotation and literature alongside, the gene lists can serve multiple computational approaches. In summary, these gene lists provide a valuable resource for researchers studying iron homeostasis in *A. thaliana*, while they also emphasize the importance of improving the accuracy and comprehensiveness of the Gene Ontology.

## Introduction

Gene Ontology (GO) is a collection of attributes (terms) with which genes are associated based on several levels of evidence (Ashburner et al., 2000). Gene Ontology is divided into the three main categories “Molecular Function,” “Cellular Component,” and “Biological Process.” While Cellular Component GO terms exclusively indicate the location of gene products from the tissue down to the subcellular location, Molecular Function GO terms describe the specific molecular activity of gene products without referencing the location, biological background, or conditions under which these activities occur. Therefore, depending on the research question, the main category Biological Process may be more relevant, in which the GO terms describe overarching processes that are usually accomplished by the interplay and/or interaction of several distinct gene products. GO terms are hierarchically organized in an upside-down tree-like structure where the upper “parent” terms are more generic and the lower “child” terms are the more specific ones.

Different GO terms are linked if a relationship exists between them. In the QuickGo database, possible relationships between GO terms include “is a,” “is part of,” “regulates,” “positively regulates,” “negatively regulates,” “occurs in,” “capable of,” and “capable of part of” (Binns et al., 2009). However, there may be variations in these relationships depending on the specific database. GO terms may also be viewed as linked containers, which include all the genes that are associated with this term. Therefore, genes associated with a GO term are often designated as “in” this term. Entries in the Gene Ontology are based on evidence codes such as “Inferred from Sequence or structural Similarity” (ISS), “Inferred from Sequence Orthology” (ISO), “Inferred from Electronic Annotation” (IEA), “Inferred from Direct Assay” (IDA), “Inferred from Mutant Phenotype” (IMP), “Inferred from Expression Pattern” (IEP), and many more (Gene Ontology, C., 2023a). We focus on a Gene Ontology’s Biological Process category. Possible relationship types of genes within this category, are “involved in,” “acts upstream of or within,” “acts upstream of or within, positive effect,” “acts upstream of or within, negative effect,” “acts upstream of,” “acts upstream of, positive effect,” or “acts upstream of, negative effect” (Gene Ontology, C., 2023b). Since GO terms have been set up to be consistent across several organisms, including plants, and curated for a plethora of different species, the GO annotations for single species are not always comprehensive. Therefore, depending on the currently available evidence for that species and the time that passes between periodic updates, GO terms may contain one or more genes or they can still be empty.

GO has been widely used for enrichment analysis of transcriptomic or proteomic data. This is possible due to the fact that the number of genes in a GO term can be used in conjunction with the total number of known genes and the respective number of regulated genes or proteins for statistical evaluation and to obtain *p*-values that allow us to infer whether or not a GO term is enriched in a set of regulated genes or proteins. This is usually accomplished by using tests for independence, with which measured frequencies or distributions are compared with expected frequencies or distributions, such as the hypergeometric test (Hahne et al., 2008), the binomial test (Aitken and Gonin, 1936), the *χ* ^2^ test (Pearson, 1900), Fisher’s exact test (Fisher, 1935), and others (Huang da et al., 2009). Due to the relative simplicity and reliability of these tests, the hypergeometric test and Fisher’s exact test are among the most commonly used statistical tests in publicly available tools for GO term or, more generally, gene set enrichment analysis (Rivals et al., 2007; Huang da et al., 2009).

Among the most widely studied model plants, *Arabidopsis thaliana* is one of the best-annotated species in terms of gene annotations and GO terms. The Arabidopsis Information Resource (TAIR) database offers various tools for browsing or downloading ontologies and performing GO term enrichment analysis, among others (Lamesch et al., 2012). Although there are other excellent tools and databases, such as MapMan (Usadel et al., 2009) and the Kyoto Encyclopedia of Genes and Genomes (KEGG) database (Kanehisa and Goto, 2000), which are very suitable for molecular function and biochemical pathway analysis, the Gene Ontology remains the most commonly used option when analyzing biological processes. The *A. thaliana*-specific Gene Ontology database is accessible through the TAIR website (https://www.arabidopsis.org > Browse > Ontologies/Keywords) (Lamesch et al., 2012).

In the past, GO term enrichment analyses have proven to be an invaluable tool in the iron field, unearthing new insights and shedding light on previously unknown connections such as the crucial role of ribosomal proteins in the context of iron deficiency (Wang et al., 2013), the identification of overlapping gene modules in the intricate interplay between phosphate and iron homeostasis (Li and Lan, 2015), and the discovery of iron-responsive genes that also respond to synthetic community of bacterial commensals (SynCom) and coumarins (Harbort et al., 2020). Moreover, it is worth noting that stress responses often result in a significant shift in iron management (Kanwar et al., 2021), further underscoring the importance of iron in many fundamental processes in plants, such as nutrition, stress response, and development. Given these results, it is clear that further investigations into iron deficiency as well as iron excess stress using GO term enrichment analysis is imperative. They also show that it is important to have a reliable and accurate resource of iron homeostasis genes for enrichment analysis of omics datasets. We used the TAIR ontology browser to investigate the GO category Biological Process for the model plant *A. thaliana* with respect to iron homeostasis and found striking gaps and shortcomings in the listed genes that we addressed to build a new resource.

## Organization of iron homeostasis in the *A. thaliana* Gene Ontology category biological process

**Figure 1:**
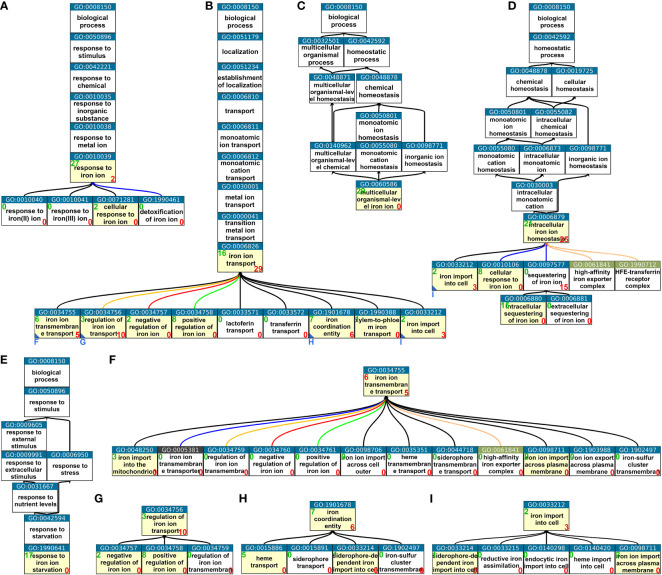
Iron homeostasis-related GO terms from the category “Biological Process.” **(A–E)** Minimal trees of Biological Process GO terms leading to the lowest possible hierarchy level with nonempty iron homeostasis-related GO terms. **(F–I)** Extensions of the minimal trees **(A–E)** where further children terms could not be displayed due to space restrictions. Small blue letters and blue triangles at the bottom left corner of a box of the minimal trees **(B**, **D)** indicate in which subfigure **(F–I)** the respective tree is completed starting with the indicated GO term. Possible children terms are not displayed if they were all empty. Yellow boxes indicate that the GO term has direct member genes, whereas white boxes indicate GO terms with no direct member genes. This does not apply to more unspecific parent terms. Green numbers at the top left corner of a box indicate the number of direct member genes. Red numbers at the bottom right corner of a box indicate the number of genes in *direct* children terms. Overlaps between direct members of a GO term and members of children terms may occur but do not necessarily have to occur. GO terms relating to iron but not iron homeostasis such as iron–sulfur cluster-related or very unspecific GO terms such as “iron binding” are not displayed. The images were created using the publicly available tool QuickGO (https://www.ebi.ac.uk/QuickGO), and the most recent Gene Ontology annotations were assessed from the TAIR database (https://www.arabidopsis.org → Browse → Ontologies/Keywords).

We utilized the Gene Ontology browser provided by the TAIR database (Lamesch et al., 2012) and the visualization tool QuickGO (Binns et al., 2009) to obtain tree images. We analyzed the minimal tree structures of iron-specific GO terms for *A. thaliana* and discovered that GO terms related to iron homeostasis were distributed among five distinct paths (**Figure 1**). The uppermost iron-specific parent GO terms of these paths were “response to iron ion” (GO:0010039) (**Figure 1A**), “iron transport” (GO:0006826) (**Figure 1B**), “multicellular organismal-level iron ion homeostasis” (GO:0060586) (**Figure 1C**), “intracellular iron ion homeostasis” (GO:0006879) (**Figure 1D**), and “response to iron ion starvation” (GO:1990641) (**Figure 1E**). In their respective paths, multicellular organismal-level iron ion homeostasis (GO:0060586) (**Figure 1C**) and response to iron ion starvation (GO:1990641) (**Figure 1E**) both contained 29 and 17 genes, respectively, with no additional genes present in any of the children terms. Thus, these were the only iron-specific GO terms in their respective paths that contained genes.

The GO term response to iron ion (GO:0010039) contained 27 genes, with two genes present in its direct child term cellular response to iron ion (GO:0071281) and no other genes in any of the other children terms (**Figure 1A**). The path leading to the GO term intracellular iron ion homeostasis (GO:0006879) was further subdivided and contained genes downstream in two more levels of children terms (**Figures 1D, I**). The largest branch was the one leading over “iron ion transport (GO:0006826). Seven direct children GO terms and eight downstream GO terms contained genes (**Figures 1B, F–I**). To summarize, the iron-specific Biological Response GO terms were organized into five distinct aspects of iron homeostasis: the general aspect of the response, the more specific aspects of the multicellular and intracellular response, the even more specific aspect of the response to iron starvation, and the most specific aspect of iron transport.

## Distribution of genes in iron homeostasis-related GO terms of the category biological process

By searching the TAIR gene ontology browser, we obtained the gene models in each iron homeostasis-specific GO term of the Biological Process category (**Figure 1**) and compiled them in the **Supplementary Tables S1**-**S19**. Although the number of splicing variants associated with iron-specific GO terms added up to a greater number, the total number of actual gene loci represented was only 113 (**Supplementary Table S1**). This resulted from multiple gene associations with different GO terms and multiple gene models for the same gene locus listed in a single GO term. For example, *FIT*, the gene of the central and essential regulator of the iron uptake machinery in *A. thaliana* (Colangelo and Guerinot, 2004; Jakoby et al., 2004; Bauer et al., 2007), was present in both response to iron ion (GO:0010039) and regulation of iron ion transport (GO:0034756) (**Supplementary Tables S5**, **S11**) while *PYE*, another regulator of iron uptake (Long et al., 2010), had multiple gene models in multicellular organismal-level iron ion homeostasis (GO:0060586) (**Supplementary Table S15**). In the following text, we repeatedly state that genes are represented in at least one of the iron homeostasis-related GO terms. These statements are meant to refer to **Supplementary Table S1**, although no reference is attached to respective statements.

Upon closer inspection of individual pairs of parent and child GO terms, we found that some genes were present in both parent and child terms, but often, genes present in the parent term were not present in the direct child term, and vice versa. For instance, a member of the YELLOW STRIPE LIKE (YSL) gene family, *YSL2*, was present in the parent term iron ion transport (GO:0006826) but not in its child term “iron ion transmembrane transport” (GO:0034755), whereas *YSL4* and *YSL6* were in the child term iron ion transmembrane transport (GO:0034755) but not in its parent term iron ion transport (GO:0006826) (**Supplementary Tables S2**, **S10**). However, *YSL2* encodes a transmembrane protein that transports iron–nicotianamine complexes (DiDonato et al., 2004), and we would thus expect it to be present in both iron ion transport (GO:0006826) and iron ion transmembrane transport (GO:0034755). Conversely, we would expect *YSL4* and *YSL6* to be found not only in the child term iron ion transmembrane transport (GO:0034755) but also in its parent term iron ion transport (GO:0006826), as they encode transmembrane proteins that export iron from chloroplasts (DiDonato et al., 2004; Divol et al., 2013). Despite this evidence having existed for many years, the information has not yet been transferred to the Gene Ontology.

One observation is that some genes may be present in iron-specific GO terms despite lacking direct evidence of involvement in iron homeostasis. For instance, the gene of a *Mitochondrial substrate carrier family protein*, AT1G07025, which has no symbol as of now, is listed in the “iron import into the mitochondrion” (GO:0034755) term with the evidence code “Inferred from Biological aspects of Ancestor.” While there is no direct evidence, other family members like MIT1 and MIT2 have been shown to transport iron into mitochondria (Jain et al., 2019). Another example is the gene *ABCB26* (AT1G70610), which is included in the iron ion transport GO term with the evidence code Inferred from Biological aspects of Ancestor and is mentioned alongside the *MIT1* gene. However, a BLAST alignment between the protein sequences of ABCB26 and MIT1 did not reveal significant similarity (data not shown), casting doubt on the involvement of *ABCB26* in iron homeostasis. Although the close similarity of a gene with others that have been demonstrated to participate in iron homeostasis may indicate a putative role in iron homeostasis or in the response to iron, the unattended automatic annotation may be prone to error. As a result, erroneous additions of genes to a GO term could affect the reliability and results of GO term enrichment analyses, particularly in GO terms with a limited number of genes.

## Representation of important regulators of iron homeostasis

We compiled a comprehensive list of all gene loci represented in iron homeostasis-related GO terms, excluding terms related to iron utilization, such as “iron-sulfur cluster assembly” (GO:0016226). The resulting list contained 113 loci (**Supplementary Table S1**) and provided lists of genes in each iron homeostasis-related GO term (**Supplementary Tables S2**-**S19**). We compared this list to the literature and found that many important genes with known functions in iron homeostasis were missing (**Table 1**). Specifically, we looked at known regulators of iron homeostasis and found that certain transcription factor genes from the bHLH IVc family, including *BHLH034* (*IDT1*) (Li et al., 2016), *BHLH104* (Zhang et al., 2015; Li et al., 2016; Wang et al., 2017), and *BHLH105* (*ILR3*) (Tissot et al., 2019; Gao et al., 2020), were not represented in iron-specific GO terms. Additionally, the gene *BHLH110*, which binds heme (Shimizu et al., 2020), was absent from the list. We also observed that repressors of iron uptake, *MYB28* and *MYB29* (Coleto et al., 2021), were not present in iron-related GO terms. Lastly, although it has been demonstrated that MYC1 interacts with FIT and negatively regulates the iron uptake machinery (Song et al., 2023), the *MYC1* gene was not present in the list.

**Table 1:** Genes with direct evidence of involvement in iron homeostasis in A. thaliana that were not represented in the Gene Ontology in any of the iron homeostasis-related GO terms but should be represented in at least one iron homeostasis-related GO term with the evidence code “Inferred from Direct Assay” (IDA) or “Inferred from Mutant Phenotype” (IMP).

AGI	Symbol or short description	Direct evidence
Metal or compound transporters		
At1g15960	NRAMP6	(Li et al., 2019)
At2g15290	PIC1/TIC21	(Duy et al., 2007)
At5g13740	ZIF1	(Long et al., 2010)
Regulators		
At3g23210	BHLH034/IDT1	(Li et al., 2016)
At4g14410	BHLH104	(Li et al., 2016; Wang et al., 2017)
At5g54680	BHLH105/ILR3	(Tissot et al., 2019)
At5g61420	MYB28/HAG1/PMG1	(Coleto et al., 2021)
At5g07690	MYB29/RAO7/PMG2	(Coleto et al., 2021)
At1g27660	bHLH110	(Shimizu et al., 2020)
At4g00480	MYC1	(Song et al., 2023)
At3g16770	ERF2	(Liu et al., 2017)
Enzymes and others		
At5g04950	NAS1	(Schuler et al., 2012)
At5g56080	NAS2	(Schuler et al., 2012)
At1g09240	NAS3	(Schuler et al., 2012)
At1g56430	NAS4	(Long et al., 2010; Schuler et al., 2012)
At1g28680	COSY	(Vanholme et al., 2019)
AT4G30190	AHA2	(Santi and Schmidt, 2009)
AT3G60330	AHA7	(Aguayo et al., 2013)

## Representation of important transporters involved in iron homeostasis

In *Arabidopsis*, transporter genes are crucial for iron homeostasis as they control the amount of iron taken up by the plant and how it is distributed or sequestered. The spatial and temporal expression of these genes are decisive in this process. Among the NATURAL RESISTANCE-ASSOCIATED MACROPHAGE PROTEIN (*NRAMP*) transporter genes, *NRAMP1*, *NRAMP3*, and *NRAMP4* (Curie et al., 2000; Lanquar et al., 2005; Cailliatte et al., 2010) were associated with iron homeostasis-related GO terms, while *NRAMP6* (Li et al., 2019) was not. Similarly, YSL transporter genes *YSL1*, *YSL2*, *YSL3*, *YSL4*, and *YSL6* (Le Jean et al., 2005; Schaaf et al., 2005; Waters et al., 2006; Conte et al., 2013; Divol et al., 2013) were included in the list, but there is a high chance that *NRAMP2* and *NRAMP5* as well as *YSL5*, *YSL7*, and *YSL8* might also be involved in iron homeostasis, given their membership in their respective paralog groups. These genes should be included in the list with the Inferred from Biological aspects of Ancestor evidence code. *ZIF1* is a direct PYE target and plays a role in nicotianamine sequestration in iron homeostasis (Long et al., 2010; Haydon et al., 2012). However, it was not included in the iron homeostasis-associated GO terms. On the other hand, genes coding for transporters of the IREG paralogs, *IREG1*, *IREG2*, and *IREG3* (Wintz et al., 2003; Schaaf et al., 2006; Kim et al., 2021), were all associated with iron-specific GO terms.

From the VIT transporter family genes, *VIT1* (Kim et al., 2006; Roschzttardtz et al., 2009), *VTL1–5* (Gollhofer et al., 2011; Gollhofer et al., 2014), and genes of the ER-body-localized VIT family members *MEB1*, *MEB2*, and AT4G27870 (Yamada et al., 2013) were in the list. However, there is no direct evidence for *VTL4* and *VTL5* and for AT4G27870. According to the TAIR ontology browser, only *VTL1*, *VTL2*, and *VTL5* were entered with the reference to direct evidence (Gollhofer et al., 2014), while the others were annotated with the evidence level Inferred from Biological aspects of Ancestor. The Zrt- and Irt-related protein (ZIP) family members and metal transporter genes *IRT1–3* (Eide et al., 1996; Lin et al., 2009; Vert et al., 2009) were present in the list. Additionally, members of the MATE transporter family are involved in iron homeostasis, and the three known genes of the citrate efflux transporter gene *MATE43/FRD3* (Roschzttardtz et al., 2011), the GOLGI-located iron transporter gene *MATE48* (Seo et al., 2012), and the gene *MATE52* (Wang et al., 2016) were included in iron homeostasis-related GO terms. *OPT3* is essential for the phloem loading of iron (Zhai et al., 2014), and the gene is also represented in the Gene Ontology. Finally, the gene for the coumarin efflux transporter *PDR9* (Rodríguez-Celma et al., 2013; Fourcroy et al., 2014) was included in the list. Among mitochondrial iron transporter genes, *NAP14* (Voith von Voithenberg et al., 2019) was present, but *PIC1* (Duy et al., 2007) was absent. *MTP8*, which mediates iron redistribution during seed development and germination (Chu et al., 2017; Eroglu et al., 2017), was also represented in GO.

## Representation of enzymes and other genes with important functions in iron homeostasis

Several genes involved in iron homeostasis encode enzymes in biosynthetic pathways of compounds required for the solubilization or transport of iron, while others function in the reduction of Fe and/or other heavy metals. However, some of the well-known genes involved in iron homeostasis, such as *NAS1–NAS4* (Schuler et al., 2012), were not represented in the list. Among the coumarin biosynthesis genes, *F6’H1* (Schmid et al., 2013; Schmid et al., 2014), *S8H* (Rajniak et al., 2018; Siwinska et al., 2018; Tsai et al., 2018), and *CYP82C4* (Rajniak et al., 2018) were present, but *COSY* (Vanholme et al., 2019) was not. *FRO2*, a well-known member of the iron uptake machinery in *Arabidopsis* (Robinson et al., 1999), was present in iron-specific GO terms, but other ferric chelate reductases were absent. However, other FRO genes are potentially involved in iron homeostasis, as *FRO3* was found to be induced under iron deficiency in Arabidopsis (Mukherjee et al., 2006), and *FRO4* and *FRO5* were demonstrated to be regulated by FIT in conjunction with the bHLH Ib transcription factors bHLH38/39/100/101 (Cai et al., 2021). Additionally, *FRO3* is a direct target of PYE (Long et al., 2010). Therefore, we suggest that the FRO family should be entirely represented in at least one of the iron homeostasis-related GO terms.

Although the phytochelatin synthase genes *PCS1* and *PCS2* have been annotated in TAIR in conjunction with Fe(III), there is no evidence that they play a role in iron homeostasis, and they have not been listed among the iron homeostasis-related genes in GO. The ubiquitin E3 ligases that interact with and target other regulators of the BHLH transcription factor family for degradation, such as *BTS* (Long et al., 2010; Selote et al., 2015), *BTSL1*, and *BTSL2* (Hindt et al., 2017; Rodríguez-Celma et al., 2019; Lichtblau et al., 2022), were also represented in the list of genes in iron homeostasis-related GO terms. In iron uptake, one of the concerted steps is the solubilization of iron by acidification of the rhizosphere by a plasma membrane H^+^-ATPase. In *Arabidopsis*, this is accomplished by *AHA2*, which is induced by iron deficiency (Santi and Schmidt, 2009). Iron deficiency-induced upregulation of another plasma membrane H^+^-ATPase gene family member, *AHA7*, implies that it might also participate in iron homeostasis but is not responsible for rhizosphere acidification (Santi and Schmidt, 2009). However, none of them was represented in iron homeostasis-related GO terms.

## Representation of FIT-regulated genes

Next, we investigated the genes that are regulated by FIT (Colangelo and Guerinot, 2004; Mai et al., 2016a) and found that some of them were and others were not represented in iron homeostasis-related GO terms (**Table 2**). Among the FIT-regulated genes according to Colangelo and Guerinot (2004), *ATAVT6B* (putative amino acid transporter), *ATL35*, *GRF11*, *CYP71B5*, *GSTL1*, *FOLB1*, *PME41*, *AT3G61930* (hypothetical protein), *MWL-2*, *SAUR18*, *AT1G14190* (glucose-methanol-choline (GMC) oxidoreductase family protein), *AT1G73120* (F-box/RNI superfamily protein), *UGT72E1*, *AT3G07720* (kelch-repeat protein), *HMA3*, *COPT2*, and *MTP3/MTPa2* were not represented in iron-specific GO terms.

**Table 2:** FIT-regulated and iron deficiency-induced genes that were not represented in the Gene Ontology in at least one iron homeostasis-related GO term but should be represented in at least one iron homeostasis-specific GO term with the evidence code “Inferred from Expression Pattern” (IEP).

AGI	Symbol or short description
FIT-regulated according to Colangelo and Guerinot (2004) (Colangelo and Guerinot, 2004)	
At1g14190	GMC oxidoreductase family
At1g34760	*GRF11/RHS5/GF14 OMICRON*
At1g73120	F-box/RNI superfamily protein
At3g07720	kelch repeat protein
At3g11750	*FOLB1*
At3g46900	*COPT2*
At3g50740	*UGT72E1*
At3g51200	*SAUR18*
At3g53280	*CYP71B5*
At3g58810	*MTP3/MTPa2*
At3g61930	hypothetical protein
At4g02330	*PME41*
At4g09110	*ATL35*
At4g19370	*MWL-2*
At4g30120	*HMA3*
At5g02780	*GSTL1*
At5g38820	putative amino acid transporter
FIT-regulated according to Mai et al. (2016) (Mai et al., 2016a) that are not mentioned above	
At1g09560	*GLP5*
At1g11080	*scpl31*
At1g14182	*SCRL28*
At1g14185	Glucose-methanol-choline (GMC) oxidoreductase family protein
At1g32380	*PRS2*
At1g53635	unknown protein
At2g35850	transmembrane protein
At4g17680	SBP (S-ribonuclease binding protein) family protein
At5g45105	*ZIP8*
At5g46060	spastin, putative; DUF599
At5g55250	*IAMT1*
At5g59520	*ZIP2*
At5g62420	NAD(P)-linked oxidoreductase superfamily protein

From the genes that were additionally found to be regulated by FIT according to Mai et al. (2016a), the positively FIT-regulated genes *GLP5*, *SCRL28*, *AT1G14185* (GMC oxidoreductase family protein), *PRS2*, *AT1G53635* (hypothetical protein), *AT2G35850* (transmembrane protein), *AT4G17680* (*S*-ribonuclease-binding protein), *ZIP8*, *AT5G46060* (spastin, putative), *IAMT1*, and *AT5G62420* (NAD(P)-linked oxidoreductase superfamily protein) were not found in any of the iron homeostasis-related GO terms. The robustly FIT-repressed genes *ZIP2* and *SCPL31* were also not present. From the most robustly iron deficiency-induced genes (Ivanov et al., 2012; Mai et al., 2016b) that have not been mentioned above, *AT3G06890* (transmembrane protein) was not represented in any of the iron homeostasis-specific GO terms.

## Representation of FIT-regulated metal transporter genes

Remarkably, the above-mentioned FIT-regulated genes that were not represented in any of the iron homeostasis-related GO terms (**Table 2**) include metal transporters known to transport other metals or putatively transport heavy metals, such as MTP3/MTPa2. Due to the low specificity of IRT1, which transports zinc and cobalt besides iron (Korshunova et al., 1999), MTP3 mediates the exclusion of zinc and cobalt from the shoot under Fe deficiency (Arrivault et al., 2006). This clearly shows that transporters not transporting iron but other metals may play important roles in the context of iron homeostasis in Arabidopsis and probably contribute to an increase in the chance of survival under iron deficiency. Such genes should be included at least in the GO term “response to iron starvation.”

The fact that *MTP3* is regulated by FIT underlines its importance. Such crosstalk between Fe homeostasis and other metals is also exemplified by the finding that with *COPT2* and the FERRIC REDUCTASE OXIDASE (FRO) gene family members *FRO4* and *FRO5*, copper uptake is induced by FIT in conjunction with the Ib transcription factors BHLH038, 39, 100, and 101 due to increased demand for copper under iron deficiency (Cai et al., 2021). It has been hypothesized that CuSOD can functionally replace FeSOD under low iron conditions (Waters et al., 2012). Likewise, the potential roles of ZIP transporters under iron deficiency have been demonstrated (Yang et al., 2010). Among the ZIP transporter genes, *ZIP2* is negatively and *ZIP8* is positively regulated by FIT (Mai et al., 2016a). *HMA3* is a member of the HEAVY METAL ATPASE (HMA) family of genes. *HMA3* is also regulated by FIT (Colangelo and Guerinot, 2004), and it was demonstrated to contribute to cobalt, cadmium, zinc, and lead tolerance by sequestration of the metals into the vacuole (Morel et al., 2009).

## The lower evidence codes if direct experimental evidence is lacking

Many genes in the TAIR-hosted Gene Ontology database were found to have been entered with the evidence code “Inferred from Biological aspects of Ancestor” (IBA). Moreover, there are more evidence codes that can be applied if direct evidence is lacking, such as ISO, ISS, or IEP. Automatic unattended entries are often useful and increase the speed by which new entries are made in Gene Ontology. However, some of the entries may be erroneous, as exemplified further above, and some prominent gene families from which several members are involved in iron homeostasis in *A. thaliana* as demonstrated in the literature, were not represented in any of the iron homeostasis-specific GO terms. Besides the NAS ortholog group, which was missing entirely, members of the YSL gene family (*YSL5*, *YSL7*, and *YSL8*) were absent in iron homeostasis GO terms. Additionally, we could not find all members of the NRAMP transporter family (*NRAMP2*, *NRAMP5*, and *NRAMP6*).

Furthermore, members of the FRO gene family (*FRO1* and *FRO3–8*) were not represented, although there is evidence for some of them that they play important roles, at least in the context of iron starvation (Waters et al., 2012; Cai et al., 2021). Besides *COPT2*, one of the FIT-regulated genes, there is evidence that the two COPPER TRANSPORTER (COPT) genes, *COPT1* and *COPT3*, influence iron homeostasis (Perea-Garcia et al., 2020). However, none of the applicable evidence codes have been used to enter these genes into any of the iron homeostasis-specific GO terms. We strongly suggest that one of these codes are applied to the above-mentioned genes, if applicable, to make the iron homeostasis-related GO terms more complete, and reliable. We suggest to not only add the missing genes but also their respective ortholog/paralog groups to the Gene Ontology with the evidence code Inferred from Biological aspects of Ancestor, Inferred from Sequence Orthology, or Inferred from Sequence or structural Similarity (**Table 3**).

**Table 3:** Genes from gene families that were not represented in iron homeostasis-related GO terms but should be represented in at least one such term with the evidence code “Inferred from Biological aspects of Ancestor” (IBA), “Inferred from Sequence Orthology” (ISO), “Inferred from Sequence or structural Similarity” (ISS), or “Inferred from Expression Pattern” (IEP), where applicable.

AGI	Symbol or short description
YSL gene family	
At3g17650	*YSL5*
At1g65730	*YSL7*
At1g48370	*YSL8*
NRAMP gene family	
At1g47240	*NRAMP2*
At4g18790	*NRAMP5*
At1g15960	*NRAMP6*
FRO gene family	
At1g01590	*FRO1*
At1g23020	*FRO3*
At5g23980	*FRO4*
At5g23990	*FRO5*
At5g49730	*FRO6*
At5g49740	*FRO7*
At5g50160	*FRO8*
COPT gene family	
At5g59030	*COPT1*
At3g46900	*COPT2*
At5g59040	*COPT3*
At2g37925	*COPT4*
At5g20650	*COPT5*
At2g26975	*COPT6*

## Gene groups and families with a high likelihood of finding new iron homeostasis-relevant members

Iron homeostasis in *A. thaliana* has been the subject of extensive research. Notable progress has been made in the sub-field of iron homeostasis regulation, particularly in the roots (Liang, 2022; Li et al., 2023). However, the mechanisms of iron homeostasis in the shoot are not yet well understood. This includes the translocation, partitioning, and redistribution of iron from source to sink organs at different developmental stages, the role of transporters and enzymes, and the regulatory mechanisms involved. As there are gene families and ortholog/paralog groups whose members have been demonstrated to participate in iron homeostasis, or that have been shown to participate in homeostasis of other transition metals, we want to point out some of the ones that are interesting to consider in further studies.

We propose the family of ZIP transporter genes as a potential candidate for further investigation in iron homeostasis. In particular, *ZIP2* and *ZIP8* have been reported to be negatively and positively regulated by FIT (Mai et al., 2016a), respectively, and *ZIP9* was induced under iron-deficient conditions and combined iron and zinc deficiency in roots (Yang et al., 2010). Although none of these genes were represented in any of the iron homeostasis-related GO terms, they may still play a crucial role in shoot iron homeostasis. Another group of interest is the small ZINC-INDUCED FACILITATOR (ZIF) and zinc-induced facilitator-like (ZIFL) transporter family, with *ZIF1* being a direct PYE target that affects the intracellular distribution of iron (Long et al., 2010; Haydon et al., 2012). However, *ZIF1* was not represented in any iron homeostasis-related GO terms.

The METAL TOLERANCE PROTEIN (MTP) transporter family includes *MTP8/MTPc3*, which participates in intracellular iron and manganese distribution in roots and shoots, is induced under iron deficiency, and its expression is FIT-dependent (Colangelo and Guerinot, 2004; Yang et al., 2010; Chu et al., 2017; Eroglu et al., 2017). *MTP3/MTPa2*, a vacuolar zinc transporter gene, is also regulated by FIT and belongs to the most robustly iron deficiency-induced genes (Colangelo and Guerinot, 2004; Mai et al., 2016a). While *MTP8/MTPc3* was represented in iron homeostasis-related GO terms, *MTP3/MTPa2* was not.

The oligopeptide transporter (OPT) family includes *OPT3*, which loads iron into the phloem and is required for shoot-to-root iron signaling (Zhai et al., 2014; Khan et al., 2018). From the Multidrug and Toxic Compound Extrusion (MATE) transporter family, *MATE43/FRD3*, *MATE48/BCD1*, and *MATE52/ESL1* play roles in iron homeostasis in *A. thaliana* (Roschzttardtz et al., 2011; Seo et al., 2012; Wang et al., 2016), and they were represented in iron homeostasis GO terms. The ATP-BINDING CASSETTE subfamily G (ABCG) transporter gene family, including the subfamily of PLEIOTROPIC DRUG RESISTANCE (PDR) transporter genes, is another interesting gene family. In particular, *ABCG37/PDR9* was demonstrated to be essential for exporting coumarins into the rhizosphere, where they enhance the mobilization of iron from the soil, especially at high pH (Rodríguez-Celma et al., 2013; Fourcroy et al., 2014; Schmid et al., 2014; Rajniak et al., 2018).

Finally, the diverse group of HEAVY METAL-ASSOCIATED (HMA) H^+^-ATPase transporter proteins, partly members of the Heavy metal transport/detoxification superfamily protein (HMP) superfamily, includes *HMA3*, which is regulated by FIT and induced under iron deficiency (Colangelo and Guerinot, 2004). Although the direct involvement of *HMA3* in iron homeostasis has not yet been demonstrated, its FIT-dependent induction under iron deficiency implies its potential importance in this context.

We estimate that there is a high chance of finding the abovementioned or other members of the *ZIP*, *ZIF*, *ZIFL*, *MTP*, *OPT*, *MATE*, *ABCG*, *PDR*, *HMA*, and *HMP* gene families/orthologs/paralogs to be crucial in the context of iron homeostasis in general or specifically in response to iron starvation, or in the response to excess iron (for which there is no GO term as of now), even if they might act on other metals or in the transport of other chemical compounds.

## Entries not present in their most appropriate GO terms

Some genes related to iron homeostasis were not represented in the most appropriate GO terms. For instance, *BTSL1* and *BTSL2* were categorized under iron ion transport (GO:0006826) and “regulation of iron ion transport” (GO:0034756) with the evidence code “inferred from mutant phenotype” and the relationship type “acts upstream of or within.” However, they were not categorized under “negative regulation of iron ion transport” (GO:0034757), which is what they have been reported to do (Rodríguez-Celma et al., 2019). Similarly, *FIT* was not classified under iron ion transport (GO:0006826), where it acts as a regulator. Instead, it was placed under regulation of iron ion transport (GO:0034756), which fits the function of FIT (Colangelo and Guerinot, 2004; Jakoby et al., 2004), but not in its most appropriate GO term, “positive regulation of iron ion transport” (GO:0034758).

*IMA/FEP* peptide genes were categorized under their most appropriate GO term, positive regulation of iron ion transport (GO:0034758), but they were not present in iron ion transport (GO:0006826), where they act as upstream regulators (Grillet et al., 2018; Gautam et al., 2021). Interestingly, *BTSL1* and *2* were the only genes categorized under “iron import across plasma membrane” (GO:0098711), but genes like *IRT1* or *FRO2*, which are directly involved in iron uptake, were not present. The relationship type acts upstream of or within is also equally valid for all the genes encoding transcription factors involved in the regulation of iron transmembrane transporters, but they were not categorized as such.

The genes mentioned above illustrate that the exact location of genes in the tree of connected GO terms and the completeness of their individual entries are crucial. As of now, the way iron homeostasis-related GO terms were populated with genes heavily impacts the detection of enrichment of the biological process of iron homeostasis as relevant GO terms are underpopulated or empty. This decreases the chances of detecting terms and increases the chances of falsely detecting terms if they have only one or very few members.

## Custom sets of genes for enrichment analysis of iron homeostasis

As almost all of the genes currently represented in any of the iron homeostasis-related Biological Process GO terms somehow participate in the response to iron ion, they should at least be listed in that particular GO term (GO:0010039). However, since this only applies to 48 of the 113 genes represented, we suggest manually performing enrichment analysis with a custom list of genes that includes all 113 previously represented genes (**Supplementary Table S20**). To make the gene set more complete, we also suggest extending the list with missing genes for which there is direct evidence of iron homeostasis participation (**Table 1**), resulting in a more complete set (**Supplementary Table S21**, “extended gene list 1”).

Although crosstalk between iron and other metals is important and some genes have been shown to be regulated by FIT with potential functions in the context of iron homeostasis, none of these genes were represented in iron homeostasis-related GO terms. Therefore, we would expect them to be included at least in the generic terms “response to iron ion” (GO:0010039) and “response to iron ion starvation” (GO:1990641), at least with the evidence code IEP or others if applicable. To account for this, we created an even further extended gene set (**Supplementary Table S22**, “extended gene list 2”), containing the genes from the previous set (**Supplementary Table S21**, “extended gene list 1”) and all FIT-regulated genes that were not previously represented in one of the iron homeostasis-related GO terms (**Table 2**).

We also suggest representing the smaller paralog/ortholog groups/families in iron homeostasis-related GO terms (**Table 3**), and we have created another further extended list that contains these genes (**Supplementary Table S23**, “extended gene list 3”) in addition to the ones in the previous list (**Supplementary Table S22**, “extended gene list 2”). Finally, we suggest adding the larger gene families that may have further yet unknown participants in iron homeostasis in the broadest sense (**Table 4**) and have assembled an experimental list that includes all of the previously mentioned gene lists (**Supplementary Table S23**, extended gene list 3) and the gene families from (**Table 4**).

**Table 4:** Gene families from which we estimate more members might be detected to play roles in the context of iron homeostasis in the future based on the fact that they transport transition metals or that known members transport other chemical compounds that are in the context of iron homeostasis.

Family	AGI locus identifiers (symbols)			
ZIF/ZIFL	AT2G48020 (*ZIF1*)	At3G43790 (*ZIF2*)	AT5G13740 (*ZIFL1*)	AT5G13750 (*ZIFL2*)
MTP	AT2G46800 (*MTP1*) AT3G61940 (*MTP2*) AT3G58810 (*MTP3*)	AT2G29410 (*MTP4*) AT3G12100 (*MTP5*) AT2G47830 (*MTP6*)	AT1G51610 (*MTP7*) AT3G58060 (*MTP8*) AT1G79520 (*MTP9*)	AT1G16310 (*MTP10*) AT2G39450 (*MTP11*) AT2G04620 (*MTP12*)
OPT	AT5G55930 (*OPT1*) AT1G09930 (*OPT2*) AT4G16370 (*OPT3*)	AT5G64410 (*OPT4*) AT4G26590 (*OPT5*)	AT4G27730 (*OPT6*) AT4G10770 (*OPT7*)	AT5G53520 (*OPT8*) AT5G53510 (*OPT9*)
MATE	AT2G04040 (*MATE1*) AT2G04080 (*MATE2*) AT2G04050 (*MATE3*) AT2G04070 (*MATE4*) AT2G04090 (*MATE5*) AT2G04100 (*MATE6*) AT1G64820 (*MATE7*) AT1G66780 (*MATE8*) AT1G66760 (*MATE9*) AT1G15150 (*MATE10*) AT1G15160 (*MATE11*) AT1G15170 (*MATE12*) AT1G15180 (*MATE13*) AT1G71140 (*MATE14*)	AT2G34360 (*MATE15*) AT5G52450 (*MATE16*) AT1G73700 (*MATE17*) AT3G23550 (*MATE18*) AT3G23560 (*MATE19*) AT1G33100 (*MATE20*) AT1G33110 (*MATE21*) AT1G33090 (*MATE22*) AT1G33080 (*MATE23*) AT3G03620 (*MATE24*) AT5G17700 (*MATE25*) AT5G10420 (*MATE26*) AT5G65380 (*MATE27*) AT5G44050 (*MATE28*)	AT3G26590 (*MATE29*) AT5G38030 (*MATE30*) AT1G12950 (*MATE31*) AT1G23300 (*MATE32*) AT1G47530 (*MATE33*) AT4G00350 (*MATE34*) AT4G25640 (*MATE35*) AT1G11670 (*MATE36*) AT1G61890 (*MATE37*) AT4G21903 (*MATE38*) AT4G21910 (*MATE39*) AT3G21690 (*MATE40*) AT3G59030 (*MATE41*) AT1G51340 (*MATE42/FRDL*)	AT3G08040 (*MATE43/FRD3*) AT2G38330 (*MATE44*) AT4G38380 (*MATE45*) AT2G21340 (*MATE46*) AT4G39030, (*MATE47*) AT1G58340 (*MATE48*) AT4G23030 (*MATE49/NIC1*) AT5G52050 (*MATE50*) AT4G29140 (*MATE51/NIC4*) AT5G19700 (*MATE52/ELS1*) AT2G38510 (*MATE53*) AT1G71870 (*MATE54/NIC2*) AT5G49130 (*MATE55/NIC3*) AT4G22790 (*MATE56*)
ABCG/PDR	AT2G39350 (*ABCG1*) AT2G37360 (*ABCG2*) AT2G28070 (*ABCG3*) AT4G25750 (*ABCG4*) AT2G13610 (*ABCG5*) AT5G13580 (*ABCG6*) AT2G01320 (*ABCG7*) AT5G52860 (*ABCG8*) AT4G27420 (*ABCG9*) AT1G53270 (*ABCG10*) AT1G17840 (*ABCG11*)	AT1G51500 (*ABCG12*) AT1G51460 (*ABCG13*) AT1G31770 (*ABCG14*) AT3G21090 (*ABCG15*) AT3G55090 (*ABCG16*) AT3G55100 (*ABCG17*) AT3G55110 (*ABCG18*) AT3G55130 (*ABCG19*) AT3G53510 (*ABCG20*) AT3G25620 (*ABCG21*) AT5G06530 (*ABCG22*)	AT5G19410 (*ABCG23*) AT1G53390 (*ABCG24*) AT1G71960 (*ABCG25*) AT3G13220 (*ABCG26*) AT3G52310 (*ABCG27*) AT5G60740 (*ABCG28*) AT3G16340 (*ABCG29/PDR1*) AT4G15230 (*ABCG30/PDR2*) AT2G29940 (*ABCG31/PDR3*) AT2G26910 (*ABCG32/PDR4*) AT2G37280 (*ABCG33/PDR5*)	AT2G36380 (*ABCG34/PDR6*) AT1G15210 (*ABCG35/PDR7*) AT1G59870 (*ABCG36/PDR8*) AT3G53480 (*ABCG37/PDR9*) AT3G30842 (*ABCG38/PDR10*) AT1G66950 (*ABCG39/PDR11*) AT1G15520 (*ABCG40/PDR12*) AT4G15215 (*ABCG41/PDR13*) AT4G15233 (*ABCG42/PDR14*) AT4G15236 (*ABCG43/PDR15*)
HMA	AT4G37270 (*HMA1*) AT4G30110 (*HMA2*)	AT4G30120 (*HMA3*) AT2G19110 (*HMA4*)	AT1G63440 (*HMA5*) AT4G33520 (*HMA6*)	AT5G44790 (*HMA7*) AT5G21930 (*HMA8*)
HMP	AT1G01490 (*HMP01*) AT1G06330 (*HMP02*) AT1G12520 (*HMP03*) AT1G22990 (*HMP04*) AT1G23000 (*HMP05*) AT1G29000 (*HMP06*) AT1G29100 (*HMP07*) AT1G30473 (*HMP08*) AT1G51090 (*HMP09*) AT1G56210 (*HMP10*) AT1G57780 (*HMP11*) AT1G63440 (*HMP12*) AT1G63950 (*HMP13*) AT1G66240 (*HMP14*) AT1G71050 (*HMP15*) AT2G18196 (*HMP16*)	AT2G28090 (*HMP17*) AT2G28660 (*HMP18*) AT2G35730 (*HMP19*) AT2G36950 (*HMP20*) AT2G37390 (*HMP21*) AT3G02960 (*HMP22*) AT3G05220 (*HMP23*) AT3G05920 (*HMP24*) AT3G06130 (*HMP25*) AT3G21490 (*HMP26*) AT3G24450 (*HMP27*) AT3G25855 (*HMP28*) AT3G48970 (*HMP29*) AT3G53530 (*HMP30*) AT3G56240 (*HMP31*) AT3G56891 (*HMP32*)	AT4G08570 (*HMP33*) AT4G10465 (*HMP34*) AT4G16380 (*HMP35*) AT4G23882 (*HMP36*) AT4G27590 (*HMP37*) AT4G33520 (*HMP38*) AT4G35060 (*HMP39*) AT4G38580 (*HMP40*) AT4G39700 (*HMP41*) AT5G02600 (*HMP42*) AT5G03380 (*HMP43*) AT5G05365 (*HMP44*) AT5G17450 (*HMP45*) AT5G19090 (*HMP46*) AT5G24580 (*HMP47*) AT5G26690 (*HMP48*)	AT5G27690 (*HMP49*) AT5G37860 (*HMP50*) AT5G44790 (*HMP51*) AT5G50740 (*HMP52*) AT5G60800 (*HMP53*) AT5G63530 (*HMP54*) AT5G66110 (*HMP55*) AT4G13380 (*MEE56*) AT5G52750 (-) AT5G48290 (-) AT3G07600 (-) AT3G04900 (-) AT1G49420 (-) AT5G14910 (-)

There is a partial overlap between the HMA and HMP families of genes. In both cases, all AGI locus identifiers are in the respective lists so they appear twice. Genes that were already represented in iron homeostasis-related GO “Biological Process” terms are included.

These lists can be used to perform enrichment analysis of iron homeostasis using simple 2 × 2 contingency table tests for independence, such as Fisher’s exact test or the *χ* ^2^ test, which are easily performed and available in the most commonly used software and frameworks for statistical analyses. We performed such enrichment analyses using Fisher’s exact test. We performed the analysis with the original set of 113 genes (**Supplementary Table S1**) and with our extended list 2 (**Supplementary Table S22**). This was applied to genes induced by iron deficiency *vs*. sufficient iron supply in the roots of 6-week-old *Arabidopsis* plants (Mai et al., 2016b). We showed that our list performed better than the original list since more genes are detected. Furthermore, enrichment was revealed with a lower *p*-value and a higher odds ratio (**Supplementary Table S24**).

## Conclusions and prospects

We investigated the iron homeostasis-specific GO terms from the Biological Process category in the *Arabidopsis thaliana* Gene Ontology database hosted by TAIR, excluding the iron–sulfur cluster and similar GO terms. We observed that a total of 113 loci were represented in 17 such GO terms that were organized into five distinct paths starting from the root of the Biological Process category and covering different aspects of iron homeostasis. Comparison of the genes represented in the relevant GO terms with the current literature revealed that important players were not represented, although evidence for their roles in iron homeostasis has been published for years. This included iron transporters *NRAMP6*, *PIC1/TIC21*, and *ZIF1*, transcription factors such as *BHLH034/IDT1*, *BHLH104*, *BHLH105/ILR3*, *MYB28/HAG1/PMG1*, *MYB29/RAO7/PMG2*, *BHLH110*, *MYC1*, and *ERF2*, and important enzymes such as the whole *NAS* ortholog group (*NAS1-4*), *COSY*, *AHA2*, and *AHA7* (**Table 1**). Furthermore, many of the FIT target genes were also not present in the iron homeostasis-related GO terms (**Table 2**). However, it can be assumed that their functions may be important in the context of iron homeostasis, as they are regulated by the central and essential transcription factor of the iron uptake machinery in roots.

It is not surprising that new findings take time to be transferred from the literature to databases like the Gene Ontology, particularly if they require human assistance. However, there were entries with evidence from the literature as recent as 2022 (**Supplementary Table S1**), while the four NAS genes, which have been known to participate in iron homeostasis for over a decade (Schuler and Bauer, 2011), were not included. This inconsistency highlights one of the main challenges of the Gene Ontology: limited coverage. We also found that some genes were represented in iron homeostasis-related GO terms but not in their most appropriate terms, such as *IRT1* and *FRO2*, which reveals another challenge: some terms in the Gene Ontology can be vague or ambiguous, making it difficult to accurately annotate genes. Additionally, we noticed that while there is a GO term for “response to iron ion starvation” (GO:1990641), there is currently no GO term covering the response to excess iron. This further highlights the shortcomings of the Gene Ontology, specifically inconsistencies in GO terms and an incomplete representation of biological processes. Finally, the Gene Ontology can be biased towards well-studied genes and processes. Additionally, there may be large gaps in knowledge transfer, possibly due to different sources of data and annotation practices, which is particularly evident in the field of iron homeostasis in *A. thaliana*. Taken together, these limitations can lead to underrepresentation or overrepresentation of certain genes and terms in the Gene Ontology and can ultimately lead to erroneous results, such as false positives and false negatives, in GO term enrichment analyses.

In order to address the limitations of the current Gene Ontology in representing the complex biological process of iron homeostasis in *A. thaliana*, we have compiled a series of comprehensive lists of genes that can be used for enrichment analysis in transcriptomic or proteomic studies. Our lists include genes with varying levels of evidence, ranging from those with direct experimental evidence of participation in iron homeostasis to those with potential roles in this process that are yet to be fully characterized.

Our most extensively validated list (**Supplementary Table S20**) includes all genes represented in iron homeostasis-specific terms in the Gene Ontology (**Supplementary Table S1**) as well as all those with direct evidence of participation in iron homeostasis in *A. thaliana* that were not previously represented (**Table 1**). Our more inclusive lists additionally incorporate genes that are known to be regulated by FIT or are robustly induced by iron deficiency (**Table 2**), which were surprisingly absent from the Gene Ontology (**Supplementary Table S21**), and missing members of small gene families or ortholog/paralog groups (**Table 3**), which could have easily been added to the Gene Ontology with the appropriate evidence codes (**Supplementary Table S22**). These lists provide a more comprehensive view of the genes involved in iron homeostasis in *A. thaliana* and highlight the gaps in the current Gene Ontology. Finally, we have further included a list of gene families of which we anticipate that members may have important roles in iron homeostasis but are currently underrepresented in the Gene Ontology (**Table 4**). This “experimental” gene list (**Supplementary Table S23**) provides an interesting resource for future studies in this field.

Overall, our lists can be used in transcriptomic and proteomic studies to perform custom enrichment analyses of iron homeostasis, which are easily performed with the most commonly used software for statistical analyses (example: **Supplementary Table S24**). Additionally, they can be used to simply look up all the hitherto known iron homeostasis-related genes in a set of genes without having to go through them manually. They provide a valuable tool for researchers studying iron homeostasis in *A. thaliana* and highlight the need for continued efforts to improve the completeness and accuracy of the Gene Ontology.

## Author contributions

H-JM and DB wrote the manuscript, H-JM and DB collected and assembled data, PB reviewed the manuscript and provided funding.
